# Interprofessional primary care during COVID-19: a survey of the provider perspective

**DOI:** 10.1186/s12875-020-01366-9

**Published:** 2021-02-03

**Authors:** Catherine Donnelly, Rachelle Ashcroft, Nicole Bobbette, Christine Mills, Amanda Mofina, Todd Tran, Kyle Vader, Ashley Williams, Sandeep Gill, Jordan Miller

**Affiliations:** 1 Queen’s University, 31 George Street, Kingston, Ontario K7L 3N6 Canada; 2 University of Toronto, 246 Bloor Street, Toronto, Ontario M5S 1V4 Canada; 3 Centre for Addiction and Mental Health, 1001 Queen St. West, Toronto, Ontario M6J 1H4 Canada; 4 Association of Family Health Teams of Ontario, 400 University Avenue, Suite 2100, Toronto, Ontario M5G 1S5 Canada

**Keywords:** Primary care teams, interprofessional primary care, COVID-19

## Abstract

**Background:**

Interprofessional primary care (IPC) teams provide comprehensive and coordinated care and are ideally equipped to support those populations most at risk of adverse health outcomes during the COVID-19 pandemic, including older adults, and patients with chronic physical and mental health conditions. There has been little focus on the experiences of healthcare teams and no studies have examined IPC practice during the early phase of the COVID-19 pandemic. The objective of the study was to describe the state of interprofessional health provider practice within IPC teams during the COVID-19 pandemic.

**Methods:**

Observational cross-sectional design. A web-based survey was deployed to IPC providers working in team-based primary care clinics in the province of Ontario, Canada. The survey included 26 close-ended and six open-ended questions. Close-ended questions were analyzed using descriptive statistics. Content analysis was used to analyze the open-ended questions.

**Results:**

445 surveys were included in the final analysis. Service delivery shifted from in-person care (77% pre-COVID-19) to telephone (76.5% during the COVID-19 pandemic). Less than half of the respondents (40%) reported receiving any training for virtual delivery. Wait times to access team members were reported to have decreased. There has also been a shift in what IPC providers report as the most commonly seen conditions, with increases in visits related to mental health concerns, acute infections (including COVID-19), social isolation, and resource navigation. Respondents also reported a reduction in healthcare provision for multiple chronic conditions including diabetes, cardiovascular disease, and chronic pain.

**Conclusions:**

IPC teams are rapidly shifting their practice to supporting their patients during the pandemic. A surge in mental health issues has been seen and is expected to continue to increase in response to COVID-19. Understanding early experiences can help plan for future pandemic waves.

## Background

Canada and countries around the globe have recognized interprofessional primary care (IPC) teams as a crucial approach to support the increasingly complex health care needs of populations [1–4]. IPC teams are defined as two or more health professionals working collaboratively to provide comprehensive, patient-centred care [4]. Team based primary care offers increased access to a range of IPC providers beyond physician only primary care, including social workers, pharmacists, dietitians, occupational therapists and physical therapists and others [1–4]. Given this breadth of perspectives, primary care teams are well placed to address the profound clinical, behavioral, and mental health care demands that are emerging and will continue to emerge throughout the pandemic [5].

The early COVID-19 research has focused on the acute care management and experiences almost exclusively from the perspective of physicians [5, 6]. Less emphasis has been placed on understanding the impact of the pandemic on non-physician IPC providers [7], despite the increasing recognition of the value of teams to manage the complexities of COVID-19 [5]. There is currently no research to provide guidance to IPC providers and teams operating during COVID-19 [7], compounding already high levels of stress experienced by healthcare providers in this context. Examining the practices and experiences of IPC providers during the early phases of the COVID-19 pandemic will inform how teams address the rapidly evolving needs of patients and communities and provide a benchmark in which to examine this evolution. The objective of this study was to describe the state of IPC provider practice within primary care teams during the COVID-19 pandemic.

## Methods

### Study design

An observational cross-sectional study design was used. A web-based survey was distributed to non-physician IPC providers working in team-based primary care clinics in the province of Ontario, Canada to answer the following questions: How are IPC providers currently providing services during the COVID-19 pandemic? How has the method of service delivery changed since the implementation of COVID-19 physical distancing requirements? How confident are IPC providers with alternate methods of service delivery being implemented during the COVID-19 pandemic? What types of patient-care issues are being seen during COVID-19 and how are these different than prior to COVID-19? What is the personal and professional impact of COVID-19 on IPC providers?

The web-based survey was developed for the study using Qualtrics (Qualtrics. Provo, UT, USA. 2013) and was informed by previous surveys of occupational therapy [8] and social work [9] practices in IPC teams in the province of Ontario, Canada. The survey was developed in collaboration with our partner organization, The Association of Family Health Teams of Ontario. All members of the research team reviewed and revised survey questions to ensure clarity. The final web-based survey included 26 close-ended and six open-ended questions that aligned with study objectives. The final survey was pilot tested with the clinical research team members, representing multiple health professions who were not involved in the initial creation of the surveys. See supplemental file 1 for a copy of the survey.

Ethics approval and consent was obtained from the Queen’s University Health Sciences and Affiliated Teaching Hospitals Research Ethics Board in Kingston, Canada (Approval # 6026691 - REH-750-19).

### Sample

We used a convenience sampling technique. The study population included non-physician IPC providers who were able to complete a web-based survey in English, and self-reported as being currently employed within a Family Health Team in the province of Ontario, Canada. Ontario Family Health Teams provide services to approximately 25% of the population in Ontario [10]. While the exact composition and number of providers varies between Family Health Teams, each team provides comprehensive care and a broad range of physical, behavioral, and mental health services [11–14]. Family Health Teams are aligned with the broader framework of the Patient’s Medical Home [4, 13].

### Recruitment

IPC providers were invited to participate in the web-based survey through recruitment emails and posts on social media (Facebook, Twitter, LinkedIn). Emails were distributed by a number of provincial professional associations. Recruitment emails and social media provided a brief description of the research project and a link to the survey. The survey link took interested participants to the first page of the survey, which contained a more detailed description of the study and a statement asking participants for consent to participate. The web-based survey was open for 15 days from April 22, 2020 until May 8, 2020. During this timeframe a state of emergency was declared in the province of Ontario, lasting from March 17th until May 19th and only essential businesses remained open and hospitals were closed to everything but urgent and emergency care.

### Data analysis

Survey data were exported from Qualtrics to Microsoft Excel. Close-ended survey questions were imported from Excel to SPSS. Data were cleaned and analyzed by five authors (NB, JM, CM, AM, TT) using descriptive statistics. Data from open-ended questions (“What is your experience during COVID-19”; “Prior to COVID-19, what were the three most common health conditions you were seeing?”; “Since COVID-19, what are the three most common health conditions you are seeing?”) were exported to Microsoft Excel and multiple authors completed an inductive content analysis for each open ended question. Authors met and compared responses until consensus was reached [16]. For the content analysis of the experience question each of six authors (RA, NB, CD, CM, KV, AW) completed a preliminary read of the first 100 responses and developed an initial coding structure that included 12 broad codes. Each author was assigned a section of the data and applied the coding structure to the responses. More than one code could be applied to a response if there were multiple statements or elements in a response. Responses were further identified as being positive, negative or neutral. Once the initial coding process was completed the six authors met to review the coding structure and ten coded responses were randomly selected and reviewed by the team to ensure the accuracy of the applied codes. Further discussion resulted in the unanimous agreement of the removal of three codes (time use, patient experience and intersection of family and work) and integrated into five themes. Authors reviewed and recoded their assigned responses in accordance with the final coding structure. For the content analysis of the conditions seen, two authors (TT, JM) independently coded each response based on health condition listed and met to compare and reach consensus on the coding of each condition listed.

## Results

A total of 473 IPC providers consented to participate in this survey, of the total 2423.48 full time equivalent IPC providers, for a response rate of 20%. Of those that consented, 445 IPC providers completed at least one survey question and were retained for full analysis of the data provided. Respondents were from twelve professional disciplines, with social workers (25%) being the largest proportion followed by dietitians (22%) and nurses (12%). Table 1 provides an outline of the demographics of the participants.

**Table 1:** Participant Demographics

Profession (*n* = 443)	Frequency (%)
Chiropodist	11 (2.5)
Dietitian	98 (22.1)
Health promotor	9 (2.0)
Mental Health Counsellor (other than social work)	12 (2.7)
Nurse	54 (12.2)
Nurse Practitioner	33 (7.4)
Occupational therapist	39 (8.8)
Pharmacist	46 (10.4)
Physiotherapist	7 (1.6)
Psychologist	2 (0.5)
Respiratory Therapist	5 (1.1)
Social Worker	110 (24.8)
Other	17 (3.8)
FTE (*n* = 408)	
0.2 - < 0.5	40 (9.8)
0.5 - < 1.0	129 (31.6)
1.0	237 (58.1)
> 1.0	2 (0.5)
Type of Employment (*n* = 420)	
Permanent	393 (93.6)
Contract	23 (5.5)
Temporary/other	4 (1.0)
Number of Years at Family Health Team (*n* = 419)	
< 1	42 (10.0)
1–2	77 (18.4)
3–4	66 (15.8)
5–6	55 (13.1)
> 6	179 (42.7)
Covid-19 Assessment Centre (*n* = 431)	
Yes	195 (45.2)

Since COVID-19, the majority of providers did not report a change in their work hours (81.5%); however, 20.5% of IPC providers were redeployed to an alternate organization or job. Table 2 provides details related to workload, referrals patterns and wait lists. Almost half (48.3%) of respondents reported experiencing greater team collaboration since COVID-19, with collaboration most frequently supported by email and phone. Table 3 outlines the nature and extent of team collaboration.

**Table 2:** Workload, Referrals and Wait times

	Frequency (%)	Experience working in primary care during COVID-19
Change in Hours since Covid-19 (*n* = 417)		
Yes-Increased	41 (9.8)	“I am working from home, and working 6–7 h/day 5 days/week, when my contract is only 3 days per week. I am on the phone almost all day every day supporting patients who are extremely anxious, depressed and scared by the COVID pandemic.”
Yes-Decreased	36 (8.6)	“Extremely quiet as very few actual patients in the building and staff hours on rotation.”
No	340 (81.5)	“I’ve been fortunate to immediately been able to work remotely at home, maintaining my hours and patient-contact.”
Received Virtual care training (*n* = 387)		
Yes	155 (40.1)	“There was a document formulated by a FHT chiropodist to help guide telephone visits which has been very helpful.”
No		“No initial training in tele health therefore having to figure it out for myself.”
Would have liked to receive additional training (*n* = 376)		
Yes	216 (57.4)	“It’s been a massive learning curve that I don’t personally or professionally feel prepared for.”
Redeployed (*n* = 386)		
Yes	79 (20.5)	“I am a screener on a Family Health Team 1 day.” “Role has been redefined to assist with the pandemic ie swabbing for disease.”
Continuing to provide services to patients with a diagnosis of COVID-19? (*n* = 384)		
Yes	57 (14.8)	
Service type change since COVID-19? (*n* = 361)		
Yes	233 (64.5)	“I have had to advocate for my role to change to be more of a check-in with vulnerable people.”
Change in referral pathway (*n* = 364)?		
Yes	57 (15.7)	
Nature of Referral Pathway (*n* = 366)		
First contact (direct patient booking)	48 (13.1)	“I have taken over managing my own referrals so do all the first contact calls, scheduling, forwarding resources, scheduling follow-ups, etc.”
Referral from most responsible provider	287 (78.4)	“I have no referrals because the physicians are not seeing as many people.”
Referral from nursing or other health care provider	9 (2.5)	
Other	22 (6.0)	“We are doing calls to the wait list. New referrals are being contacted within 2–4 wks for a triage call.”
Wait times prior to Covid-19 (*n* = 365)		
Same day	28 (7.7)	
Less than 1 week	81 (22.2)	
1–2 weeks	91 (24.9)	
> 2–4 weeks	71 (19.5)	
> 4–6 weeks	50 (13.7)	
> 6–8 weeks	17 (4.7)	
> 8–10 weeks	8 (2.2)	
> 10–12 weeks	19 (5.2)	
Wait time since Covid-19 (*n* = 362)		
Same day	73 (20.2)	“Referrals have dramatically dropped. Many patients have preferred to postpone their follow-ups until in-person appointments are available again.” “Significant reduction in referral volume as primary care physicians are seeing less patients than prior to COVID-19.” “More consults to follow-up with patients.” “Still busy and demand for services.”
Less than 1 week	116 (32.0)	
1–2 weeks	84 (23.2)	
> 2–4 weeks	47 (13.0)	
> 4–6 weeks	12 (3.3)	
> 6–8 weeks	7 (1.9)	
> 8–10 weeks	5 (1.4)	
> 10–12 weeks	18 (5.0)

**Table 3:** Teamwork and Collaboration

	Frequency (%)	Question #32: What is your experience working in primary care during COVID-19
Impact on Collaboration (*n* = 385)		
Less collaboration	129 (33.5)	“We generally have frequent team meetings or connect with each other informally, which has not been happening nearly as much.”
Same amount of collaboration	70 (18.2)	“Routine check in and encouragement are crucial for the morale of the team.”
More collaboration	186 (48.3)	“It has solidified the team as a whole.”
How are you collaborating with your team?		
By phone	302 (19.8)	“We had a lot of support with daily team phone calls as we all got started and figured things out.”
By video	258 (16.9)	“We have greatly reduced team meetings which are now over video.”
By email	311 (20.4)	“I receive some email updates but not a lot.”
By text messaging	187 (12.2)	
In-person in clinic	108 (7.1)	
Through EMR communications	322 (21.1)	
Social media	20 (1.3)	
Other	20 (1.3)	

Prior to COVID-19, IPC providers reported an average of nearly 70% of their time was spent delivering direct in-person care. On average, the majority of the care provided to patients prior to COVID-19 occurred in-person at the clinic (77.0%) and in-person at their patients’ home (21.4%). Since COVID-19 IPC providers reported that an average of 61% of their time was spent delivering one-on-one care, with the majority of care having shifted to telephone-based encounters (76.5%), followed by some in-person care in the clinic (27.3%). Table 4 provides details on the delivery of IPC services.

**Table 4:** Delivery of Interprofessional Primary Care Services

	Mean % (SD)
Percent of time delivering patient care prior to COVID-19	
Direct care delivered 1:1 (*n* = 404)	69.3 (16.0)
Direct care delivered in a group (*n* = 270)	14.3 (14.2)
Indirect Care (n = 404)	24.3 (17.3)
Percent of time delivering patient care since COVID-19	
Direct care delivered 1:1 (*n* = 394)	61.5 (24.4)
Direct care delivered in a group (*n* = 103)	9.2 (12.7)
Indirect Care (n = 394)	38.7 (25.1)
Percent of time spent in delivering direct patient care prior to Covid-19	
In-person (clinic) (*n* = 369)	77.0 (22.7)
In-person (home) (*n* = 139)	21.4 (27.7)
In-person (public space) (*n* = 46)	15.5 (23.7)
Phone (*n* = 355)	16.6 (19.2)
Video (*n* = 65)	6.9 (17.8)
Email (*n* = 199)	6.8 (9.8)
Text (*n* = 47)	3.7 (4.8)
Social media (*n* = 22)	1.8 (2.9)
Other (*n* = 15)	18.9 (30.0)
Percent of time spent in delivering direct patient care since Covid-19	
In-person (clinic) (*n* = 115)	27.3 (27.9)
In-person (home) (*n* = 32)	12.8 (22.7)
In-person (public space) (*n* = 17)	6.9 (16.9)
Phone (n = 384)	76.5 (24.9)
Video (*n* = 155)	18.1 (21.6)
Email (*n* = 224)	11.2 (11.2)
Text (n = 43)	4.8 (4.1)
Social media (*n* = 30)	9.9 (19.4)
Other (*n* = 16)	26.3 (36.3)
Percent confidence in care delivery method	
In-person (clinic) (*n* = 332)	94.5 (14.0)
In-person (home) (*n* = 233)	80.7 (29.3)
In-person (public space) (*n* = 172)	63.9 (34.2)
Phone (n = 387)	81.4 (17.5)
Video (*n* = 331)	68.6 (25.8)
Email (*n* = 284)	56.5 (32.1)
Text (*n* = 162)	40.7 (33.9)
Social media (*n* = 119)	30.8 (31.4)
Other (*n* = 20)	17.5 (36.1)

**Fig. 1:**
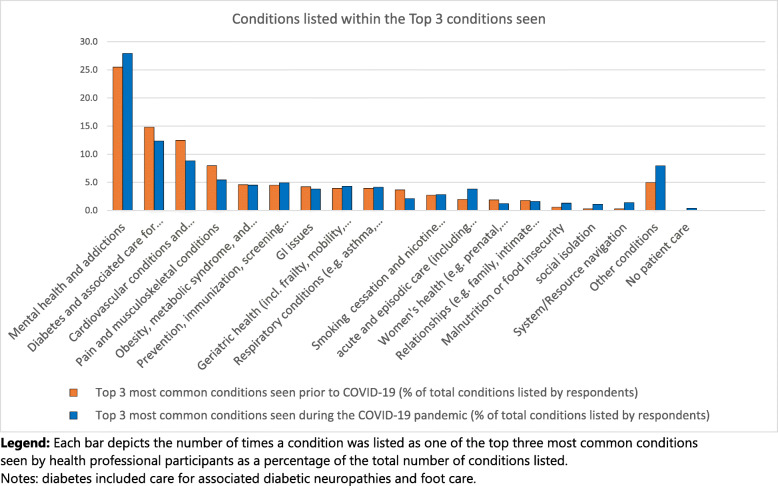
Top three most frequently seen conditions prior to and during the COVID-19 pandemic. Legend: Each bar depicts the number of times a condition was listed as one of the top three most common conditions seen by health professional participants as a percentage of the total number of conditions listed. Notes: diabetes included care for associated diabetic neuropathies and foot care. Cardiovascular conditions included care for risk factors for cardiovascular conditions, such as hyperlipidemia and hypertension. Prevention, immunization, screening included routine immunizations, well baby visits, and other routine screening. Acute and episodic care included infections such as upper respiratory infections and COVID-19. Women’s health visits included visits for prenatal, postnatal, pelvic health, and menopause. System/resource navigation included navigation of financial resources

The most common conditions identified when participants were asked to list the three most common health conditions they were seeing prior to COVID-19 were mental health or addictions concerns (25.5% of all conditions listed within the top three most frequency seen conditions were mental health or addictions), diabetes (14.8%), cardiovascular conditions (12.5%), and pain or musculoskeletal conditions (8.0%). See Table 5 and Fig. 1 for the full results. During the COVID-19 pandemic, there was an increase in the frequency with which acute or episodic care, including upper respiratory infections and COVID-19, were reported within the top three most commonly seen conditions (3.8% of conditions listed in the top three during COVID-19 vs. 2.0% prior to COVID-19). Additionally, four other conditions were reported more frequently (> 0.5% increase) within the three most common health conditions seen during the COVID-19 pandemic: mental health and addictions concerns (26.9% during COVID-19 vs. 25.5% prior to COVID-19), malnutrition and food insecurity (1.3% during COVID-19 vs. 0.6% prior to COVID-19), social isolation (1.1% during COVID-19 vs. 0.3% prior to COVID-19, health system or resource navigation (1.4% during COVID-19 vs. 0.3% prior to COVID-19). There were several conditions that were reported less frequency (> 0.5% decrease) within the three most common health conditions seen during the COVID-19 pandemic: diabetes (12.4% during the pandemic vs. 14.8% prior to COVID-19), cardiovascular conditions (8.8% during the pandemic vs. 12.5% prior to COVID-19), pain and musculoskeletal conditions (5.4% during the pandemic vs. 8.0% prior to COVID-19), cognitive issues including dementia (2.1% during the pandemic vs. 3.7% prior to COVID-19), women’s health appointments (1.2% during the pandemic vs. 1.9% prior to COVID-19).

**Table 5:** Most common conditions seen prior to COVID-19 and during the COVID-19 pandemic

	Most common health conditions seen				Top 3 most common health conditions seen			
	What was the most common health condition you were seeing Pre-COVID19? (n of responses)	% of respondents	What is the most common health condition you are seeing during the COVID19 pandemic? (n of responses)	% of respondents	Number of times these conditions were listed within the top 3 most common conditions seen Pre-COVID19	% of total responses	Number of times these conditions were listed within the top 3 most common conditions during the COVID19 pandemic	% of total responses
Mental health and addictions	100	27.2	109	30.4	272	25.5	275	26.9
Anxiety	59		72		82		99	
Depression	19		7		80		70	
Depression/anxiety	7		5		13		10	
Stress	1		2		18		17	
Trauma/PTSD	1		1		19		12	
Addictions/substance use	1		0		6		3	
Personality disorder	0		1		5		4	
“mental health”	13		21		39		44	
other	0		1		11		14	
Diabetes and associated care for diabetic neuropathies (e.g. foot care)	96	26.1	76	21.2	158	14.8	123	12.4
Cardiovascular conditions and addressing cardiovascular risk	28	7.6	17	4.7	133	12.5	88	8.8
Pain and musculoskeletal conditions	20	5.4	16	4.5	85	8.0	54	5.4
Obesity, metabolic syndrome, and weight management	25	6.8	15	4.2	49	4.6	45	4.5
Prevention, immunization, screening (e.g. developmental assessment and ‘well baby’ visits)	19	5.2	15	4.2	48	4.5	49	4.9
GI issues	3	0.8	7	2.0	45	4.2	38	3.8
Geriatric health (e.g. frailty, mobility, falls)	18	4.9	19	5.3	42	3.9	43	4.3
Respiratory conditions (e.g. asthma, COPD)	14	3.8	12	3.4	42	3.9	41	4.1
Cognitive issues (e.g. dementia, learning disabilities)	16	4.3	12	3.4	39	3.7	21	2.1
Smoking cessation and nicotine addition	8	2.2	10	2.8	29	2.7	28	2.8
acute and episodic care (e.g infections such as acute upper respiratory infections and COVID19)	10	2.7	23	6.4	21	2.0	38	3.8
Women’s health (e.g. pre/post natal, pelvic health, menopause)	4	1.1	2	0.6	20	1.9	12	1.2
Relationships (e.g. family, intimate partner)	0	0.0	1	0.3	19	1.8	16	1.6
Malnutrition or food insecurity	0	0.0	1	0.3	6	0.6	13	1.3
Social isolation	0	0.0	1	0.3	3	0.3	11	1.1
System navigation	0	0.0	3	0.8	3	0.3	14	1.4
Other conditions	7	1.9	15	4.2	53	5.0	79	7.9
No patient care	0	0.0	4	1.1	0	0.0	4	0.4

A total of 377 responses were received to the open-ended question “what has been your experience working in primary care during COVID-19”. Five main themes emerged from the open responses including: access, shifting roles, working in teams, personal impact and inadequate guidance. Each theme contained both positive and negative statements which was thought to reflect the wide variation in provider experiences during the first weeks of the pandemic as IPC practice shifted, almost overnight, to providing virtual care. Virtual care includes synchronous and asynchronous forms of communication such as the use of video platforms, telephone, instant messaging as well as others. The theme of ‘Access’ reflected both the increased access to services that were now available to patients by phone or video, but also a concern over barriers to access due to difficulty using technology, accessing technology or a general preference by patients to receive face-to-face care.

The theme of ‘Shifting Roles’ highlighted the changing roles that participants across each of the professions experienced in part due to the rapid shift to virtual care. Some participants indicated that they were able to maintain their roles through virtual care delivery, whereas others were unable to contribute to patient-care because their role was incompatible or difficult to implement through virtual means (e.g. home safety assessments, or specific procedures). Many of the participants attributed shifting roles to the referral process used to link patients with IPC providers. Although some participants grappled with increased work, many others described having a reduction in role that was a result of reduced physician-initiated referrals. Others took on completely new roles to support the management of COVID-19 in assessment centres or their own clinics.

‘Working in Teams’ emphasized the unique experience participants reported of working in an IPC team during the early phase of the COVID-19 pandemic. Many participants highlighted the support the team provided during the transition to virtual care, and emphasized the ability of the team to come together to solve the challenge of safely delivering care presented by COVID-19. However, many participants overwhelmingly expressed feelings of isolation because of the lack of contact with their teams and the lack of daily hallway conversations.

‘Personal Impact’ was the fourth theme and it highlighted the significant personal impact on participants working during the early phase of the COVID-19 pandemic. Participants described feelings of isolation, worry, and exhaustion. Some participants expressed that they felt a sense of greater purpose and meaning because they were able to help their colleagues and patients during the immediate COVID-19 pandemic crisis.

‘Inadequate Guidance’ was an overarching theme that highlighted a general sense that primary care had not been a focus of COVID-19 planning. IPC providers identified the initial wave of COVID-19 had focused on the medical rather than the social and emotional aspects of health and as primary care shifted to focus on emergency or urgent care IPC providers received little guidance as to where their services fit. Lack of guidance made it difficult to prioritize patient issues and more broadly to determine the role of the IPC team. Sample quotes can be found in Table 6.

**Table 6:** Experiences Working in Primary Care during COVID-19: Four Themes and Representative Quotes

Theme and Sub-themes	Sample Quotes
**Shifting Roles**	
Explicit Role Change	“I have been redeployed to the hospital so am working more in acute care. I have found though the primary care team to be less inclusive of allied health in COVID planning and decision making.” “Shift in my role to more system planning locally, COVID-19 related activities (assessment centre), Long Term Care swabbing. Less typical primary care activities (prevention etc).”
Implicit Role Change	“I have had to quickly adjust my role to meet the needs of our patients and organization at this time. Thus, my role in some ways looks different than how I provide care typically.” “More acute management versus chronic management.”
Role Halted	“There is little I can do for most of my clients via telephone other than check-in and ensure they have everything they need at home.” “Many are cancelling services for nutrition counselling.” “Leadership team has instructed to cut back on all non-essential occupational therapy services.”
No role change, modality change	“The biggest change has been working from home and not seeing patients in person. Other than that a lot of the workload is the same.”
**Access**	
Changing capacity	“With fewer non-clinical interruptions throughout the day, I’ve been able to increase my capacity for clinical care.” “I hope that moving forward we can continue to implement providing MORE care over the phone or via [videoconferencing]. I can see MORE patients during the day due to COVID with these changes of most care being provided over the phone.” “I find that I’m able to reach more pts. in a day than previously, when in person visits were scheduled. This may be d/t the fact that most pts. and caregivers are also home during the day and able to participate in call.”
Improved availability	“It has reaffirmed the importance/necessity of in-person visits for many patients. It has also opened up the door to other possibilities as phone/virtual visits are possible and potentially even easier/more accessible for some patients.” “Patients loving the phone access.” “Several patient populations such as busy moms or seniors prefer phone follow-up as saves time or does not involve driving to appts.”
Seeking health care	“I realize people are living with their ailments rather than seeking medical care immediately.”
Technology barriers	“Difficult with those who are hearing impaired like many seniors.” “Difficult to assess non-verbal when performing geriatric assessments. Difficult to assess cognition.” “Depending on age demographics of patients and their comfort with technology, I am sometimes limited in what I can accomplish over the phone.”
**Working in Teams**	
Supporting each other	“Supporting and understanding.” “Great team support.” “Our entire team work and support each other daily.”
Working together to create solutions	“The team has rallied and come together and is functioning well to ensure patients are given high quality care despite challenges.” “Overall I’d have to say it’s been a learning experience in collaboration. I find our FHT has really pulled together and offered to help each other with programs/initiatives.”
Missing team connection	“I have certainly missed the camaraderie of working as a co-located team, and I am getting fewer “quick questions” from my colleagues. “
Organizational leadership	“When collaboration amongst all providers of care from clerical to physicians and IHPs can be done the improvement seen in morale and patient care is significant. When Admin appears out of touch and not engaged with the team the negative impact is more emphasized as it is already a period of uncertainty and fear for many team members.” “I have felt incredibly supported by our management with recognition that this is different and hard and employees are managing various roles at home (not only remote working).” “It has been very challenging, lacking a lot of guidance and consistent direction from physicians/management in our office.”
**Personal Impact**	
Isolating	“Working from home has been unusual and isolating as I am accustomed to working with patients and colleagues in person.”
Uncertainty and stress	“Nothing is the same and it changes day to day.” “Worries about how COVID-19 may affect self, family and colleagues - especially having to come into the clinic during the early days of the pandemic.”
Mental and emotional toll	“It is mentally and emotionally draining, as it is so hard to be effective without face to face visits or efficient when the service delivery has change [d] so much.” “Constant COVID conversations with clients in distress constantly requires more time for self care, while balancing own family and needs.”
Meaningful	“For those families that are able to have appointments with me, I know I am making an impact.” “My experience has been very positive-life changing actually. From a personal perspective, seeing our health team band together to support our community and to be witness to it is something I have no words for.” “Honoured to be part of a Family Health Team.” “Very exhilarating -proud to be a part of this.”
**Inadequate Guidance**	
	“I feel that the hospital and media are all hyped on the hospital but everyone has forgot about primary care.” “It has been hard to triage patients to determine whether or not they are considered “emergency or urgent care” as defined by our [professional] college.” “Lack of decisions from the health system about how allied heath can support province-wide goals/plan. Individual teams/centres left to figure this out for themselves. Frustration among allied staff who do not have clear direction as to what essential service they can provide”

## Discussion

The rapidly emerging pandemic literature has focused on acute and institutional medical care [5, 7, 17]. We are not aware of any other papers that have examined IPC teams during the COVID-19 pandemic. Results of our study demonstrate the rapidly shifting roles of IPC providers and the need for immediate guidance so that primary care teams can be better prepared to care for the clinical, behavioral, and mental health needs anticipated in future COVID-19 waves.

This study was conducted in the province of Ontario, Canada, the countries most populated province [10]. This context offers an important opportunity to examine team-based care during COVID-19, with IPC teams having been in place and operating across the province for well over a decade [11]. Many countries across the globe have recognized the importance of team-based primary care [1–4] and the Family Health Teams in Ontario represents a well-established model of team based primary care [4].

Our results show the overwhelming focus on supporting mental health both before and since COVID-19. In part, this is because mental health is a prevalent issue in primary care [14] and also because interprofessional teams were conceived to support physicians in managing increasingly complex populations, specifically with regards to increasing chronic mental and physical health conditions [4, 15]. Even in the early weeks of the COVID-19 response there was an increased focus on mental health, specifically anxiety, and this was also being reported in the literature [5, 18]. There is evidence that the COVID-19 pandemic will heighten the need for acute and long-term mental health care supports for individuals and populations [5, 19]. It is being recognized by health officials internationally, nationally and provincially that the mental health impact could be as significant as the COVID-19 virus itself [5, 17–19]. The data also highlights an increase in appointments to support patients experiencing social isolation and health system navigation, which is suggestive of this growing indirect effect of COVID-19, which is expected to continue [17]. Although IPC team members are trained and experienced in providing mental health care, a significant challenge arising from the COVID-19 pandemic is to ensure these services remain accessible to patients as service delivery has rapidly shifted to virtual care [20].

While there was an increased focus on mental health, there was a shift away from supporting chronic physical health conditions. The study has highlighted that access to in-person primary care was largely halted during the early phase of the pandemic, however, there continued to be full access to virtual services from IPC providers. Our results suggest that the shift away from providing care to individuals with chronic conditions could reflect either a preference for individuals with chronic conditions to delay their appointments or targeted appointments to older adults and those known to be socially isolated or at risk of loneliness. It has been noted in the emerging COVID-19 literature that there is an expected wave of secondary health issues related to the postponement of non-urgent appointment and services [21]. Teams should consider how they can reach out to patients with chronic conditions to offer virtual supports or connect to community supports.

Primary care is unique compared to acute care settings in that their care delivery has shifted almost entirely to virtual care [20]. Prior to COVID-19 virtual care had limited uptake in primary care [20]. It is clear from the results of this survey that providers are seeing benefits to patients, families and themselves and research will need to shift to examine who most benefits from this mode of delivery and what approaches are most effective [22]. Our study was conducted in the early weeks of the COVID-19 response and emphasizes the need to integrate ongoing comprehensive planning and supports for virtual care. It will be important to conduct follow-up with these same providers at regular intervals to continue to understand the experiences and use of virtual care over time and with experience [22].

One of the challenges highlighted in the study is that the existing referral process used to engage IPC providers may not be effective at ensuring patients have full and direct access to these providers. Referrals to IPC team members are primarily through physicians, and the COVID-19 response has highlighted the need for reconsideration of this traditional referral model to improve access to the range of services embedded within interprofessional models of primary care. The literature has identified referral processes as a critical component of supporting access to and fostering integrated primary care [23, 24]. Moving forward teams need to consider how patients can have direct access to team members to best support patients managing the direct and indirect consequences of COVID-19.

Collaboration and communication within teams has been shown to be critical in supporting integrated and coordinated care [22, 23, 25]. Almost half of the teams reported increased teamwork since COVID-19 and suggest teams are building on strong processes and using their collective expertise to build solutions to support access. Strong collaborative teams have been shown to lead to positive health outcomes [26, 27] and COVID-19 has highlighted specifically the value of teams in mobilizing their resources to support their patients.

It must be acknowledged that Family Health Teams represent one model of primary care and generalization to all primary care models should occur with caution. Providers were asked to identify the most common conditions seen and this is not the same as conducting a chart audit of practice, which would provide a direct measure of practice patterns. We also recognize that the conditions seen and changes in the conditions were what were reported by participants and are not the actual prevalence of these conditions. The survey represents the state of IPC during the early phase of the COVID-19 restrictions and it is anticipated that the experience in primary care will continue to evolve. Ongoing research is needed throughout the phases of the pandemic to examine the long term and emerging impact on team-based primary care.

## Conclusion

The study provides an important picture of IPC and highlights the role that each sector of the health care system has in managing the far-reaching impacts of COVID-19. Supporting access to and awareness of IPC providers’ services will be crucial in ensuring patients receive supports they need, including direct referral pathways, and mitigating barriers to receiving virtual care.

## Data Availability

The datasets used and/or analyzed during the current study are available from the corresponding author on reasonable request.

## Abbreviations

COVID-19: coronavirus disease 2019
IPC: interprofessional primary care
